# Postcoordination of codes in ICD-11

**DOI:** 10.1186/s12911-022-01876-9

**Published:** 2022-05-17

**Authors:** Kristy Mabon, Olafr Steinum, Christopher G. Chute

**Affiliations:** 1 Canadian Institute for Health Information, 495 Richmond Road, Suite 600, Ottawa, ON K2A 4H6 Canada; 2 Nordic Centre for Classifications in Health Care, Rörviksvägen 19 SE-45197, Uddevalla, Sweden; 3 Division of General Internal Medicine, Public Health, and Nursing, Institute for Clinical and Translational Research, Johns Hopkins University, 2024 E Monument St, Suite 1-200, Baltimore, MD 21287 USA

**Keywords:** Postcoordination, Classification, ICD11, International classification of diseases

## Abstract

A new coding feature introduced with ICD-11, the 11th revision of the International Classification of Diseases (ICD), is postcoordination, which supports combining (linking) two or more codes into a cluster that describes a clinical concept. Postcoordination allows for coded data to be reported to a greater level of specificity than was possible in previous version of ICD. The linked codes are kept together in a cluster when submitted for reporting. This article presents background detail on the postcoordination feature in ICD and the postcoordination tool. Also presented are several examples that demonstrate the flexibility that ICD-11 provides for enriching coded health information.

## Background

ICD-11, the 11th revision of the International Classification of Diseases (ICD), is designed to be used for coding of diagnoses in electronic settings. Postcoordination has been newly applied to ICD-11 as a feature that supports combining (linking) two or more codes into a cluster that more richly describes a clinical concept. For morbidity reporting purposes, postcoordination allows users to code a clinical concept to its greatest level of specificity as documented by the health care practitioner.

For some clinical concepts, pertinent clinical information has been precombined in ICD-11 in a single stem code. This precombination is referred to as precoordination. For example, the clinical concept “duodenal adenocarcinoma” is classified to “2B80.00 Adenocarcinoma of duodenum.” The detail of histopathology and anatomy site has been precombined in one single code. When the details of a documented clinical concept are not reflected in a single code, postcoordination may be used to capture additional detail. Postcoordination allows for coded data to be reported to a greater level of specificity than was possible in ICD-10. The linked codes are kept together in a cluster when submitted for reporting. This article presents background detail on postcoordination, the new coding feature in ICD-11, along with several examples that demonstrate the flexibility that it provides for enriched coding of health information.

## Main text

### Functional feature of ICD-11

Postcoordination, the focus of this article, is a *functional* feature of ICD-11 rather than a *content* feature. Conceptual frameworks or models usually inform classification systems and ontologies. ICD-11 is no exception. There are indeed conceptual *models* underlying various elements of ICD-11 (e.g., 1. a 3-part framework for describing healthcare-related harms—described in an article in this series [1]; 2. a framework for categorizing different extension code types—also described in a companion article [2]; and 3. conceptual grouping of disease concepts in each of the disease content chapters of the classification). A companion article on ICD-11 architecture and structure provides more detail on conceptual elements of the classification [3]. It is noteworthy that the dagger-asterisk convention of ICD-10, a precursor to ICD-11’s postcoordination, has a conceptual model that dictates a coding practice of assigning two ICD-10 codes to link etiology (†) and manifestations (*). In consideration of ICD-10, we acknowledge that postcoordination in ICD-11 is rooted in the overriding concept that clinical diagnoses often need to be characterized further through the coding of related information on etiology, severity, laterality, manifestations, related complications, etc. Postcoordination, as described in this article, is the new feature in ICD-11 that unlocks the potential for rich characterization of clinical diagnoses in such dimensions.

### Stem codes and extension codes

Any discussion of postcoordination requires consideration of the two code types in ICD-11: stem code and extension code. Stem codes are found in the tabular list of ICD-11 and can be used alone. ‘Section X Extension Codes’ is the one section in ICD-11 in which the basis of postcoordination was envisaged since extension codes cannot be used alone. The extension codes are a special type of code that can be used to provide additional detail to a linked stem code. Extension codes provide information such as severity scale value; temporality; aetiology; topology scale value; anatomy and topography; histopathology; dimensions of injury; dimensions of external causes; consciousness; substances; diagnosis code descriptors; capacity or context; and health devices, equipment and supplies. Extension codes are described in more detail in another article in this series [2].

### Postcoordination in ICD-11

**Fig. 1:**
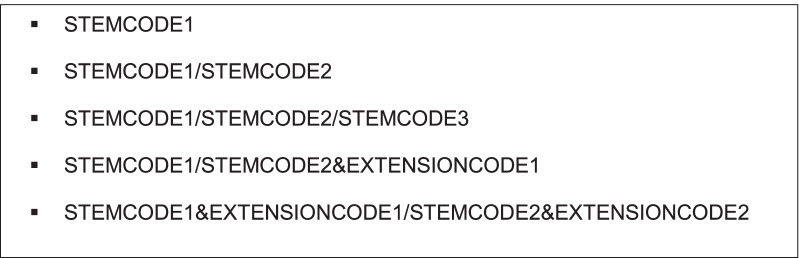
Stem codes and clusters

One of the noted benefits of ICD-11 in comparison to ICD-10 is the ability to post-coordinate codes in ICD-11 [4]. Postcoordination allows users to link core diagnostic concepts (i.e., stem code concepts), and add additional detail captured in extension codes to stem code concepts. The linked codes are referred to as a cluster and a cluster must always begin with a stem code (Fig. 1). Postcoordination allows for the capture and reporting of more precise description of the clinical diagnosis. (Information is provided below for conventions used to link codes in a cluster.)

We begin with a complicated example to demonstrate the power of postcoordination. Ensuing text explains the rules surrounding postcoordination, as well as some simpler examples. In this complex first example, we consider the code assignment for a patient with a “closed transverse intertrochanteric fracture of the right hip after a fall from tripping over a loose rug at home.” A number of individual diagnostic codes are implicated for a complete description of the injury event, and postcoordination is of great value to capture all the details. The ICD-11 stem code NC72.30 will be selected to describe the intertrochanteric hip fracture. This hip fracture stem code could be linked to the external cause of injury stem code PA60 (Unintentional fall on the same level or from less than 1 m). Each of the respective stem codes can be further refined by postcoordination of extension codes. The hip fracture stem code (NC72.30) is further specified by linking to extension codes for laterality (XK9K Right), fracture subtype (XJ5V7 Transverse fracture), and whether the fracture was open or closed (XJ44E Closed fracture). Meanwhile, the external cause of injury stem code (PA60) can be further specified by linking to extension codes for the object producing injury (XE3WK Rug, mat, loose carpet) and the place of occurrence (XE266 Home). The resulting code cluster for this complex clinical concept is NC72.30&XK9K&XJ5V7&XJ44E/PA60& XE3WK&XE266.

- Example: Closed transverse intertrochanteric fracture of the right hip after a fall from tripping over a loose rug at home.
- Cluster: NC72.30&XK9K&XJ5V7&XJ44E/PA60& XE3WK&XE266
- Code descriptions:
  - NC72.30 Intertrochanteric fracture of femur (Stem code)
  - Laterality: XK9K Right (Extension code)
  - Fracture subtype: XJ5V7 Transverse fracture (Extension code)
  - Fracture open or closed: XJ44E Closed fracture (Extension code)
  - Associated with: PA60 Unintentional fall on the same level or from less than 1 metre (Stem code)
  - Object or substance producing injury: XE3WK Rug, mat, loose carpet (Extension code)
  - Place of occurrence: XE266 Home (Extension code)

### Conventions used in postcoordination

Two specific conventions are used to separate codes in a cluster when postcoordination is involved:

1. Forward slash (/)—This is always used to separate a primary stem code from other stem codes when two or more stem codes are selected to form a cluster
  Example: Personal history of invasive ductal carcinoma of breast
  Cluster: QC40.3/2C61.0
  Code descriptions:
    QC40.3 Personal history of malignant neoplasm of breast (Stem code)
    2C61.0 Invasive ductal carcinoma of breast (Stem code).

2. Ampersand (&)—This is always used to link an extension code to a stem code or link one extension code to another extension code in a cluster. Extension codes can never be used without a stem code and thus an ampersand will always precede an extension code in a cluster. More than one extension code can be linked in a cluster.
  Example: Contusion left ear after fall downstairs in home
  Cluster: NA00.2&XK8G&XJ9NV/PG50&XE3HC&XE266
  Code descriptions:
    NA00.2 Superficial injury of ear (Stem code)
    Laterality: XK8G Left (Extension code)
    Type of injury: XJ9NV Contusion (Extension code)
    Associated with: PG50 Fall or jump of undetermined intent on the same level or from less than 1 metre
    Object or substance producing injury: XE3H Stairs, step (Extension code)
    Place of occurrence: XE266 Home (Extension code)

More examples of postcoordination in the morbidity use case are shown in Table 1. Examples of postcoordination in the quality and safety use case are shown in another article in this series [1].

**Table 1:** Examples of postcoordination in the morbidity use case

Example 1	Acute ST elevation myocardial infarction, anterior wall, LAD Cluster: BA41.0&XA7RE3&XA7NQ7 BA41.0 Acute ST elevation myocardial infarction Specific anatomy: XA7RE3 Anterior wall of heart Specific anatomy: XA7NQ7 Left anterior descending coronary artery
Example 2	Acute pyelonephritis, left side, E. coli Cluster: GB51&XK8G&XN6P4 GB51 Acute pyelonephritis Laterality: XK8G Left Infectious agent: XN6P4 Escherichia coli
Example 3	Diabetic coma; Type 2 Diabetes mellitus Cluster: 5A23/5A11 5A23 Diabetic coma Has causing condition (code also): 5A11 Type 2 diabetes mellitus
Example 4	Left inguinal hernia with acute obstruction Cluster: DD51&XK8G/ME24.2&XT5R DD51 Inguinal hernia Laterality: XK8GLeft Has manifestation (use additional code, if desired): ME24.2 Digestive system obstruction Course: XT5R Acute
Example 5	Concussion and open fracture shaft of left ulna due to fall on uneven sidewalk Cluster: NA07.0/PA60& XE1DA&XE53A Cluster: NC32.2 & XK8G& XJ7YM /PA60 & XE1DA & XE53A NA07.0 Concussion Associated with (use additional code, if desired): PA60 Unintentional fall on the same level or from less than 1 m Object or substance producing injury: XE1DA Uneven surface, not elsewhere classified Place of Occurrence: XE53A Sidewalk NC32.2 Fracture of shaft of ulna Laterality: XK8G Left Fracture open or closed: XJ7YM Open fracture Associated with (use additional code, if desired): PA60 Unintentional fall on the same level or from less than 1 m Object or substance producing injury: XE1DA Uneven surface, not elsewhere classified Place of Occurrence: XE53A Sidewalk
Example 6	Kaposi’s sarcoma of the soft palate in HIV disease Cluster: 2B57.Y&XA8HL5/1C62.3 2B57.Y Kaposi sarcoma of other specified primary sites Specific anatomy: XA8HL5Soft palate Associated with (use additional code, if desired) 1C62.3HIV disease clinical stage 4 without mention of tuberculosis or malaria
Example 7	Type 1 diabetes mellitus with diabetic nephropathy; Diabetic cataract Cluster: 5A10/GB61.Z Cluster: 9B10.21/5A10 5A10 Type 1 diabetes mellitus GB61.Z Chronic kidney disease, stage unspecified 9B10.21 Diabetic cataract 5A10 Type 1 diabetes mellitus
Example 8	Pneumococcal pneumonia causing sepsis Cluster: CA40.07/1G40 CA40.07 Pneumonia due to Streptococcus pneumonia 1G40 Sepsis without septic shock

Examples from the ICD-11 Reference Guide [4] (examples 1–6 from Sect. 2.11.2, examples 6–8 from Sect. 2.24.18)

### Postcoordination electronic tool in ICD-11

**Fig. 2:**
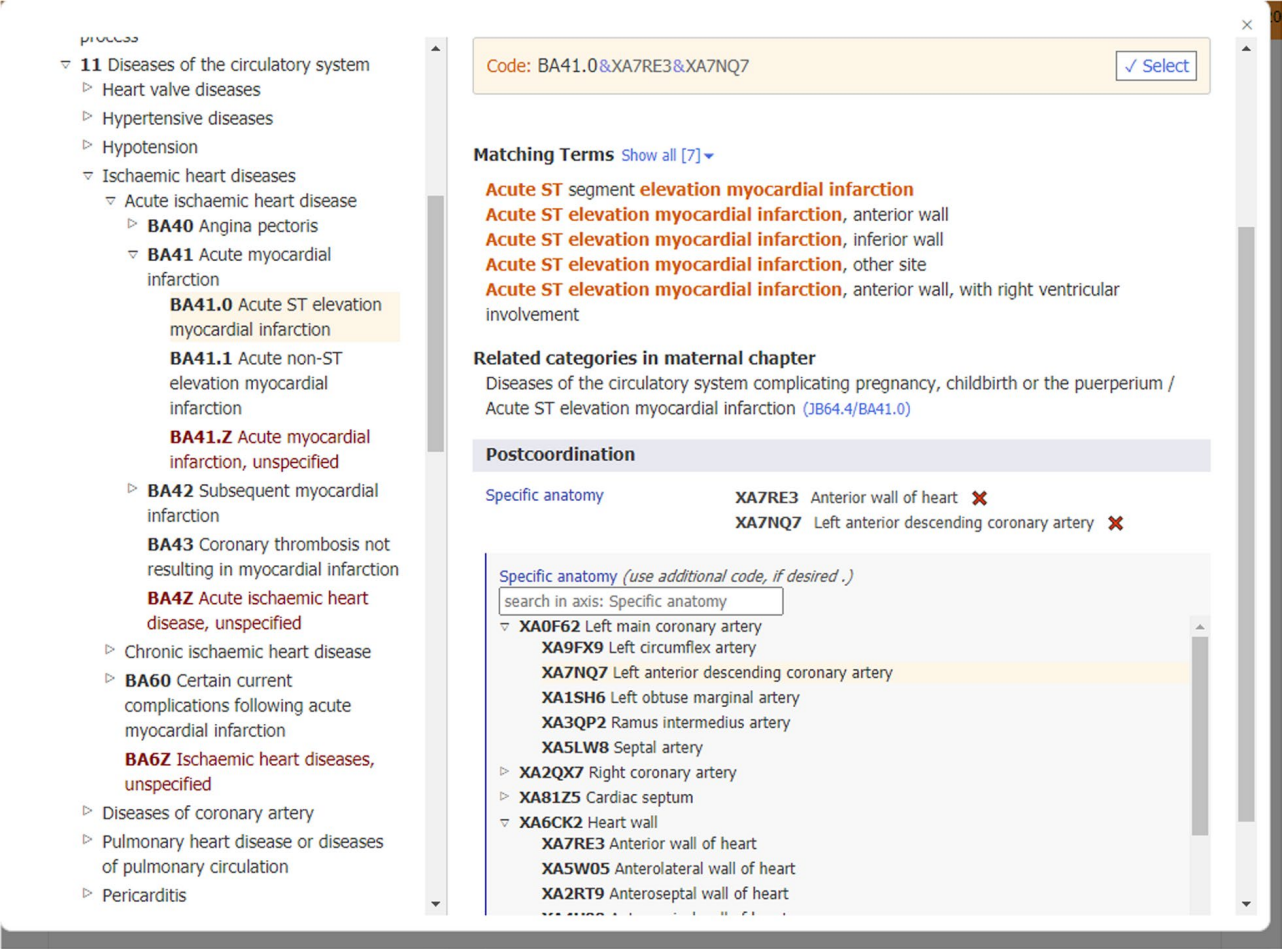
An example of how postcoordination works in ICD-11

The postcoordination tool is embedded within the online ICD-11 browser [5] and the online ICD-11 Coding Tool [6] and is separate from the ICD-11 system design. The electronic postcoordination tool supports users in finding codes to link to the chosen stem code and automatically applies the appropriate convention to link certain codes (i.e., forward slash or ampersand). The postcoordination tool appears at stem codes for which specific postcoordination axes have been set as applicable to link other codes to the stem code chosen. An example of how postcoordination works in the tooling is presented in Fig. 2.

There are instances where postcoordination is mandatory for the case being coded. Mandatory instances are identifiable within the postcoordination tool under the label “Has causing condition (code also).” This instructional note indicates to the user that they must link an additional code describing aetiological information to the stem code when that information is available. This concept is comparable to the dagger code convention in ICD-10 and appears for entities that may be caused by an underlying disease. For example, with chronic renal failure secondary to type 2 diabetes the postcoordination tool supports the user to link GB61.Z (Chronic kidney failure, stage unspecified) and 5A11 (Type 2 diabetes mellitus), creating a cluster GB61.Z/5A11. We expect one aspect of international adoption by countries is that some countries may require additional post-coordination as part of their national adoption.

A postcoordination instructional note labeled “Has manifestation (use additional code, if desired)” is a reminder to the user that, depending on the case being coded, it is allowable to post-coordinate any manifestations with the underlying condition. This instructional note appears for entities that may develop manifestations, and the listed manifestations available within the postcoordination tool are typically the most frequent manifestations resulting from the underlying condition. For example, regarding esophageal ulcer with acute hemorrhage, the postcoordination tool supports the user to link DA25.Z (Oesophageal ulcer, unspecified) with the manifestation ME24.90 (Acute gastrointestinal bleeding, not elsewhere classified), creating a cluster DA25.Z/ME24.90 [4].

An “associated with” postcoordination instructional note appears for entities where multiple codes are allowed or may be required (e.g., for specific use cases, such as three-part quality and safety model) to fully describe a distinct clinical concept and may not necessarily represent a cause/effect relationship [4].

“Sanctioning rules” embedded in the postcoordination tool help avoid incorrect or prohibited postcoordination. For example, fracture of shaft of ulna is a precoordinated concept in ICD-11; therefore, it is incorrect or prohibited to replicate the meaning of the clinical concept by choosing stem code NC32.Z (Fracture of forearm, unspecified) and extension code XA8U33 (Shaft of ulna). If the user tries to select the extension code XA8U33 with NC32.Z, the postcoordination system will automatically change the chosen stem code to NC32.2 (Fracture of shaft of ulna). However, the sanctioning rules do not prevent all instances of incorrect or prohibited use of postcoordination, and there are opportunities to enhance the rules to minimize such instances. One example is the clinical concept of diabetic retinopathy. Diabetic retinopathy is also a precoordinated concept in ICD-11 (9B71.0Z); however, depending on how the user searches the concept in the ICD-11 Coding Tool, as of 2022, they may code the concept in one of the following ways:

1. 9B71.Z (Retinopathy, unspecified) and 5A11 (Type 2 diabetes mellitus)

Cluster: 9B71.Z/5A11.

2. 9B71.0Z (Diabetic retinopathy, unspecified) and 5A11 (Type 2 diabetes mellitus)

Cluster: 9B71.0Z/5A11.

In the first instance, when the user selects 5A11 to post-coordinate with 9B71.Z, there is no sanctioning rule embedded in the system at 9B71.Z to alert the user that they have chosen the incorrect stem code for the clinical concept of diabetic retinopathy. At present, it is possible that a user may classify the concept of diabetic retinopathy in two ways, with the latter using the correct stem code for diabetic retinopathy in the cluster.

The above is a consequence of postcoordination, but one anticipated by the developers. Sanctioning rules are intended to guard against violation of the code uniqueness requirement in the ICD-11 for Mortality and Morbidity Statistics and to specify what are permissible combinations of codes. Sanctioning rules are a system of checks which are effectively large look-up tables that include precoordinated expressions and their post-coordinated equivalents.

Thus, if a post-coordinated equivalent is entered into any coding tool supported by ICD-11 applications, the user will be warned and pointed to the precoordinated equivalent. Correspondingly, if the post-coordinated expression is created manually, lists of coded diagnoses can be easily run through filters that will algorithmically replace post-coordinated expressions with their canonical precoordinated equivalents. However, the diabetic retinopathy example illustrates the sanctioning rules will need to continue to be refined.

### Postcoordination: challenges

As one of the new coding features in ICD-11, the addition of postcoordination axes at chosen stem codes will continue to expand with daily use of the classification.

Users may initially experience some challenges and limitations with using the postcoordination tool. In some instances, the user may encounter coding a clinical concept where a chosen stem code is missing a specific postcoordination axis, requiring the user to postcoordinate codes manually. For example, when coding a diagnosis of profound hearing loss, there is no postcoordination axis for severity available at the chosen stem code (AB51.Z Deafness not otherwise specified). The user may manually postcoordinate the severity extension code (XS2R Profound) with the chosen stem code and create the cluster AB51.Z&XS2R.

To rectify the above challenges and limitations, users of the ICD-11 may submit a proposal via the maintenance platform for changes to the postcoordination combination at a chosen stem code. A proposal for an update to an ICD-11 postcoordination option at a chosen stem code is considered an update at a more detailed level, and such updates can be published at annual rates. In the interim, users have the option to manually add additional codes when the postcoordination option for which the user is looking for is not available in the postcoordination tool at the chosen stem code.

The postcoordination tool places codes in a cluster in the order that the user looked for them**.** In some instances, the user may have to re-order codes created by the postcoordination tool to align with certain coding instructions on order of codes. For example, when coding a diagnosis of sepsis secondary to urinary tract infection, the coding rule in the ICD-11 Reference Guide [1] is that the type of infection must be ordered before the code for sepsis as indicated in the coding note at the sepsis code. Consideration of a future enhancement to the functionality of the postcoordination tool to allow the user the opportunity to re-order codes in a cluster to support coding instructions, when necessary, is recommended.

For certain clinical diagnostic statements, the ICD-10 encourages the use of multiple codes for reporting. With ICD-11 this instruction has been expanded and systematized through postcoordination and it offers a way to report clinical diagnoses at a detailed level for both main condition and all other conditions. The postcoordination tool embedded within the online ICD-11 browser and the online ICD-11 Coding Tool offers an easy way for the user to utilize postcoordination when coding and is but one benefit of ICD-11.

## Conclusion

In closing, the new postcoordination feature in ICD-11 has tremendous potential. This is widely recognized among the many stakeholders who have contributed to ICD-11 development and field testing. It is widely understood however, that there will be a learning curve for all stakeholders as countries move to system wide deployment. Ultimately, the new ICD-11 features, including postcoordination, are designed to enhance and refine health information to enable better health system management.

## Data Availability

All data and materials presented in this manuscript are available from the World Health Organization. ICD-11 Reference Guide. https://icd11files.blob.core.windows.net/refguide/html/index.html

## Abbreviations

ICD: International classification of diseases
ICD-10: International classification of diseases, 10th revision
ICD-11: International classification of diseases, 11th revision
